# POCUS: Presence, Observation, Connection, Understanding, Story

**DOI:** 10.24908/pocusj.v10i02.19432

**Published:** 2025-11-17

**Authors:** Sanjay A. Patel

**Affiliations:** Department of Graduate Medical Education, Riverside Methodist Hospital (OhioHealth), Columbus, OH, USA

**Keywords:** Humanism, POCUS

6:00 PM. It was pitch black. A brutally cold Chicago Winter. The end of a long, exhausting call day. Sign-out time. “This guy's probably the sickest one on the list,” my senior resident said. I was an intern. The patient—let's call him Mike—had been admitted with complications from alcohol-related cirrhosis and cardiomyopathy. His abdomen was distended, his temples sunken, and he wore the expression of someone losing a decades-long battle with alcohol.

“Hey doc, could I see what you're looking at?”

We already knew the diagnosis. The plan was in place. However, I was eager to practice point of care ultrasound (POCUS)—a skill I was beginning to learn. It wasn't going to change our decisions that day, but it gave me a reason to linger. To be curious and learn. To be at the bedside. To connect.

My resident began scanning and turned the screen toward him. I mimicked the motion of a healthy heart with my hands. Mike watched in awe as we visualized his dilated ventricle, struggling to contract. I moved the probe to his abdomen, pointing out a nodular, shrunken liver adrift in ascites. We saw clinical images; he saw his consequences. He saw his story. We performed a bedside paracentesis, draining nearly 11 liters of fluid. With every new bottle, he exclaimed, “You're still going? Sheesh!”

Mike gradually stabilized. He was seen by several specialists and eventually discharged home with cautious optimism. Over the course of the week, we had several more looks into his abdomen with the ultrasound probe. These images stayed with me as they were among my first experiences with POCUS. As the intern caring for someone without a primary care provider, I became Mike's by default. With this, he was scheduled to see me in clinic the next week for a follow-up appointment.

At that first clinic visit, he burst with energy: “I still can't believe what you showed me in the hospital, doc! I'm done! Never touching that stuff again!” I wanted to believe him—but I couldn't keep my skepticism at bay. I'd seen stories like his before in my short medical career. A decades-long addiction rarely ends after a single hospitalization.

But this time, it did. Over the next two and a half years, Mike transformed. He stopped drinking alcohol. His ascites dramatically resolved, and he was taken off diuretics. His ejection fraction improved—not normal, but better. His cheeks filled out. His energy returned. Every visit was filled with jokes and laughter. He managed to avoid readmission over those few years.

The last time I saw Mike was during my final week of residency. I brought in a new intern to transition his care. He retold his story—how he had seen his own heart and liver in that hospital bed. “That did it doc,” he said. “What you showed me made me realize.” I thought to myself, “How does that small moment change his life forever?” Those 2D grayscale videos—captured on a weathered, beaten-down ultrasound machine—shifted something inside him. A mirror held up at a moment of crisis.

And here's the thing: it didn't change our management; it changed everything. Clinically, we would've done the same. But that moment—sitting side by side, the sickest moments of his life intersecting with my early medical career—created something no medication or note could. A shared recognition. A quiet, visual turning point. A visceral truth, no longer abstract.

We often say, “POCUS changes management.” Sometimes, yes. But more often: POCUS influences. It confirms. It clarifies. It creates trust. It invites. That night, it invited Mike into the clinical space in a way that made him feel seen. And seemingly for the first time, he truly saw and understood himself. The value wasn't in image precision, quality, or diagnostic performance.

It was in presence. The simple act of being there. Of showing and seeing—together.

Fifteen years later, I still see Mike's face—not sunken or weary, but full of light. He taught me that sometimes, what a person needs most isn't more tests, more treatment, or more advice. Sometimes, the most powerful intervention is to see what we see, with someone who sees them in return.

